# Effectiveness of Insecticide Spraying and Culling of Dogs on the Incidence of *Leishmania infantum* Infection in Humans: A Cluster Randomized Trial in Teresina, Brazil

**DOI:** 10.1371/journal.pntd.0003172

**Published:** 2014-10-30

**Authors:** Guilherme L. Werneck, Carlos H. N. Costa, Fernando Aécio Amorim de Carvalho, Maria do Socorro Pires e Cruz, James H. Maguire, Marcia C. Castro

**Affiliations:** 1 Instituto de Estudos em Saúde Coletiva (UFRJ) e Departamento de Epidemiologia, Instituto de Medicina Social/IMS, Universidade do Estado do Rio de Janeiro (UERJ), Maracanã, Rio de Janeiro, Rio de Janeiro, Brasil; 2 Department of Global Health and Population, Harvard School of Public Health, Boston, Massachusetts, United States of America; 3 Instituto de Doenças Tropicais Nathan Portella and Universidade Federal do Piauí, Teresina, Piauí, Brasil; 4 Departamento de Bioquímica e Farmacologia, Centro de Ciências da Saúde, Universidade Federal do Piauí, Teresina, Piauí, Brasil; 5 Programa de Pós-Graduação em Ciência Animal, Centro de Ciências Agrárias, Universidade Federal do Piauí, Teresina, Piauí, Brasil; 6 Division of Infectious Diseases, Brigham and Women's Hospital, Harvard Medical School, Boston, Massachusetts, United States of America; The Faculty of Medicine, The Hebrew University of Jerusalem, Israel

## Abstract

**Background:**

To evaluate the effect of insecticide spraying for vector control and elimination of infected dogs on the incidence of human infection with *L. infantum*, a randomized community intervention trial was carried out in the city of Teresina, Brazil.

**Methods/Principal Findings:**

Within each of ten localities in the city, four blocks were selected and randomized to 4 interventions: 1) spraying houses and animal pens with insecticide; 2) eliminating infected dogs; 3) combination of spraying and eliminating dogs, and 4) nothing. The main outcome is the incidence of infection assessed by the conversion of the Montenegro skin test (MST) after 18 months of follow-up in residents aged ≥1 year with no previous history of visceral leishmaniasis (VL). Reactions were measured at 48–72 h, induration of ≥5 mm considered positive. Interventions were executed after the baseline interview and repeated 6 and 12 months later. The effects of each type of intervention scheme on the incidence of infection were assessed by calculating relative risks and 95% confidence intervals using Poisson population-averaged regression models with robust variance. Among the 1105 participants, 408 (37%) were MST positive at baseline. Of the 697 negatives, only 423 (61%) were reexamined at the end of the follow-up; 151 (36%) of them converted to a positive MST. Only dog culling had some statistically significant effect on reducing the incidence of infection, with estimates of effectiveness varying between 27% and 52%, depending on the type of analysis performed.

**Conclusions/Significance:**

In light of the continuous spread of VL in Brazil despite the large scale deployment of insecticide spraying and dog culling, the relatively low to moderate effectiveness of dog culling and the non-significant effect of insecticide spraying on the incidence of human infection, we conclude that there is an urgent need for revision of the Brazilian VL control program.

## Author Summary

Zoonotic visceral leishmaniasis (VL) constitutes a serious public health problem in the Americas, particularly in Brazil. The disease is caused by the protozoan parasite *Leishmania infantum*, which is transmitted by the bite of female sand flies, and dogs are the main source of infection. To decrease the risk of transmission, the Brazilian VL control program recommends residual insecticide spraying and environmental management for vector control, and culling of seropositive dogs in areas with moderate to high levels of transmission. Because there is a lack of scientific evidence supporting such interventions, we designed a study to assess the effectiveness of dog culling and residual insecticide spraying in the reduction of incidence of human VL infection. The results show that only dog culling had some statistically significant effect on reducing the incidence of infection, with estimates of effectiveness varying between 27% and 52%. In light of the continuous spread of VL in Brazil despite the large scale deployment of insecticide spraying and dog culling, the relatively low to moderate effectiveness of dog culling and the non-significant effect of insecticide spraying on the incidence of human infection, we conclude that there is an urgent need for revision of the Brazilian VL control program.

## Introduction

Zoonotic visceral leishmaniasis (VL) is a severe neglected tropical disease leading to 4.5 to 6.8 thousand new cases each year in the Americas, mainly those living in poverty [1], [2]. In this region, the disease is caused by the protozoan parasite *Leishmania infantum* (syn = *Leishmania chagasi*), which is transmitted by the bite of female sandflies from the genus *Lutzomyia*, and dogs are considered the main source of infection in urban settings [3], [4]. Those who are infected usually exhibit no symptoms, but some 5–10% will develop clinical signs of the disease during the course of infection [5], [6]. Clinical VL is commonly characterized by fever, weight loss, hepatosplenomegaly, and pancytopenia, and is usually fatal if untreated [7], [8]. Malnutrition and genetic factors may play a role in the risk of developing clinical VL after infection [5], [9], [10].

Brazil accounts for some 90% of the disease burden in the Americas, with an estimate of 4.2 to 6.3 thousand cases per year and fatality rates around 7% [1]. A gradual process of VL urbanization started in the early 1980s in Brazil, initially causing epidemics in the cities of Teresina, Natal and São Luis, all located in the Northeast of the country, and later spreading to other major urban centers [11]. Between 2008 and 2010, 11,581 autochthonous VL cases were reported in 1,392 Brazilian municipalities, with 70% of cases occurring in only 165 municipalities, which had a total population of 40 million persons and included 12 state capitals and 52 cities with >100,000 inhabitants.

Currently, the VL control program of the Brazilian Ministry of Health recommends two strategies for reducing the risk of transmission: (i) vector population control by means of residual insecticide spraying and environmental management, and (ii) culling of seropositive dogs in areas with moderate to high levels of transmission [12]. However, both strategies have proven unsuccessful in interrupting transmission [4], [13], [14]. Indeed, a systematic review of studies conducted in Latin America concluded that there is a lack of scientific evidence to support the effectiveness of such interventions [15]. Ten community intervention trials evaluated the effectiveness of dog-culling and residual insecticide spraying strategies, alone or in combination, and findings were contradictory. The authors of the review identified frequent methodological problems, such as small number of clusters for comparison, lack of comparability between groups in terms of exposure to infection, use of inaccurate diagnostic methods for detecting infection in human and dogs, small sample sizes, and high rates of loss to follow-up [15].

Because there are few alternatives for controlling zoonotic VL we attempted to address the methodological problems of previous community intervention studies and designed a cluster randomized trial to assess the effectiveness of dog culling and residual insecticide spraying in the reduction of incidence of human VL infection. The trial was conducted during the years of 2004–06 in the city of Teresina, Brazil, one of the largest endemic areas for VL in Brazil. We present our findings and conclusions following the recommendations of the updated version of the CONSORT statement [16].

## Materials and Methods

### Study site

Teresina is the capital of the State of Piauí, located in the Northeast region of Brazil at 05°05′ latitude South and 42°48′ longitude West and 339 km inland at 72 m above sea level. Its population of 814,230 inhabitants (2010) occupies an area of 1,392 km^2^ with a population density of 584.94 inhabitants/km^2^. The climate is tropical, with mean annual temperature 27°C and annual rainfall 1,300 mm. The highest temperatures occur between August and December, and the rainy period occurs from January until April. The periphery of the city has areas of pasture and tropical forest, including babassu and carnauba palm groves, with the predominant vegetation cover consisting of medium-sized bushes.

Until 1980, only sporadic VL cases had occurred in Teresina. In 1980, however, the city was the site of the first large urban epidemic of VL in Brazil [17]. From 1980 to 1985, almost 1,000 new cases were detected as the population increased from 370,000 to 460,000 inhabitants. The disease remained an important public health problem throughout the 1980s, although the incidence declined to less than 100 cases a year after 1985. There was a second epidemic of 1,200 cases between 1993 and 1995. During the first half of the 2000s, the incidence averaged approximately 20 cases per 100,000 inhabitants, and leveled off after 2005 at around 10 cases/100,000 inhabitants.

### Trial design

**Figure 1:**
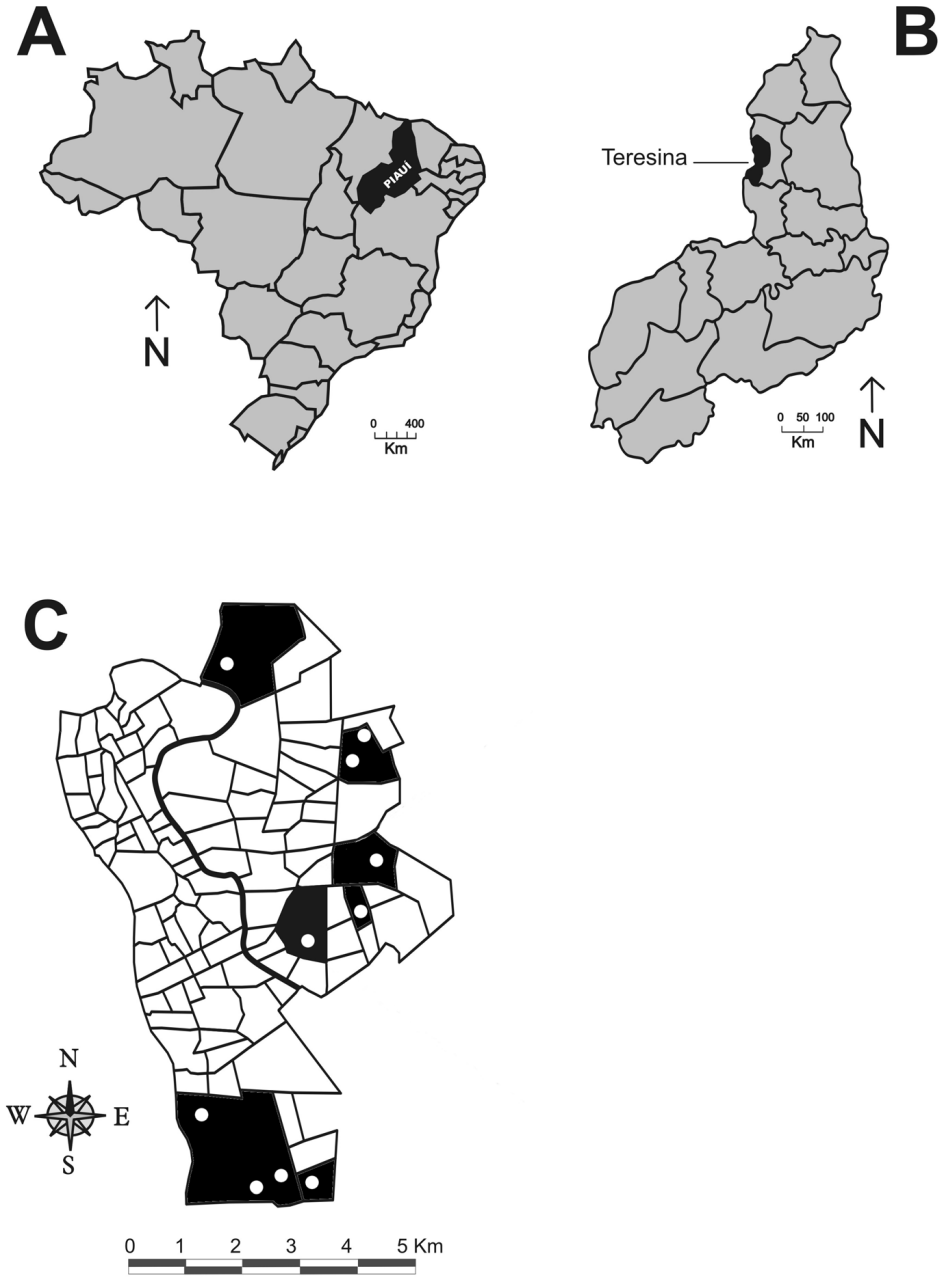
Map of the neighborhoods of the city of Teresina, Piauí. In dark are those neighborhoods selected for the study and the white dots indicate the localities within the neighborhoods in which the study was carried out.

A cluster randomized trial was carried out from January 2004 to December 2006 in ten localities (*localidades*) in seven neighborhoods (*bairros*) of the city of Teresina, that had cases of VL reported from 2000 to 2002 (Figure 1). Selection of the neighborhoods was designed to include different regions of the city, as well as a variety of land use and vegetation cover patterns.

Based on detailed sketch maps routinely utilized by vector control teams, each of the ten localities was divided into blocks, each containing an average of 60 residences. The average number of blocks per locality was 30.9 (range: 13–63), and for each locality, four blocks were selected in a stepwise fashion as follows to minimize the risk of cross-contamination of interventions in each locality: (i) the first block was selected at random; (ii) all blocks sharing a border with the first block selected were excluded from the pool of eligible blocks for selection; (iii) the second block was selected at random from the pool of eligible blocks; (iv) steps (ii) and (iii) were repeated until four blocks were selected. Figure S1. Schematic representation of the sampling process.

### Study population

Eligible participants were residents of selected blocks aged 1 year or above with no history of VL. In each block, around 25 residences were visited and one eligible person in each household was selected for the study by simple random sampling from a list of the names of the residents. Selected persons remained eligible for participation if they had no evidence of previous infection, as indicated by a negative result of a Montenegro skin test (MST) using 0.1 ml of leishmania antigen injected intradermally with reactions measured 48–72 hours later [18]. The antigen was prepared from a strain of *Leishmania amazonensis* and provided by the Reference Centre for Diagnostic Reagents (Biomanguinhos—FIOCRUZ/RJ, Brazil). The diameter of skin induration was evaluated by two experienced and extensively trained professionals. The test was considered positive when induration measure was ≥5 mm in diameter. If the selected person was absent or refused to participate (this occurred less than 5% of the time) or had a positive Montenegro reaction, the next youngest resident on the list was selected instead. At the last visit (18 months) a new MST was performed.

At the time of the initial visit, all consenting participants had blood samples collected by venipuncture in order to test for the presence of antibodies to *L. infantum* by an indirect immunofluorescent serological test (IFAT) using the Biomanguinhos—FIOCRUZ/RJ, Brazil, kit according to the manufacturer's instructions. The original plan was to repeat the IFAT test at 6 and 12 months, but due to operational problems, data on IFAT results were not considered valid for the analysis, and serology was not used as a marker of infection in the study. Problems with serology were poor sensitivity and reproducibility. For instance, among the 951 subjects for which an IFAT result was available at baseline, only 16 (1.68%) were positive. This result was deemed incompatible with the knowledge about VL transmission in Teresina, particularly in the studied areas in which transmission is known to occur, and inconsistent with data previously obtained indicating human seroprevalences ranging from 13.9% to 46.0% [19], [20]. To check whether the error was in our laboratory, 827 randomly selected sera were sent to a retest at the National VL Reference Laboratory, Fundação Ezequiel Dias (FUNED), in Belo Horizonte. Again, seroprevalence was also extremely low (1.33%) and agreement between laboratories was considered poor (kappa = 0.08). It was unclear whether the problem with serology was due to substandard techniques for handling and storage of the collected sera, problems with test execution or problems with the kit itself. In any case, we decided not to use IFAT results in this study and relied on conversion of the MST at 18 months of follow-up as the only outcome measure, since no clinical cases of VL were detected among the studied population.

Using a structured questionnaire with pre-coded questions, data were obtained on age, sex, literacy, history of migration (ever lived outside Teresina), time of residence in Teresina, number of people in household, history of VL in the family, and characteristics of the household structure, peridomestic environment, and presence of domestic animals. Written consent was obtained from all participants (or, if they were aged <18 years, written consent was obtained by one of their caregivers along with verbal assent from those above 10 years old).

### Interventions

Four interventions schemes were defined: (i) No intervention, (ii) Insecticide spraying (household and residential annexes), (iii) Culling of seropositive dogs, and (iv) Insecticide spraying+culling of seropositive dogs. Interventions were delivered in the selected blocks every 6 months, for three times, beginning just after each household visit. The last visit (18-month visit) was not followed by any intervention.

Both culling of seropositive dogs and insecticide spraying were performed according to the routine of the Visceral Leishmaniasis Control Program of the Zoonosis Control Center (ZCC) of the Teresina City Health Department. Teams of health workers of the ZCC with expertise in delivering such interventions were specifically recruited for this study. Interventions were performed in all houses of the blocks selected for receiving that specific intervention, not only in the houses where subjects had been recruited for the study.

All domiciled dogs in the blocks under the dog culling intervention had blood samples collected by venipuncture for serological testing by indirect immunofluorescent antibody test (IFAT) using a canine leishmaniasis kit supplied by Bio-Manguinhos, FIOCRUZ, Rio de Janeiro. Reactions were considered positive if promastigote membrane fluorescence was observed at a serum dilution of 1∶40. Positive sera were retested for confirmation. Dogs with a confirmed seropositive result were transported to the ZCC where they were anesthetized and killed following legal procedures [12].

Insecticide spraying was performed in all internal and external walls (up to 3 meters of height) of households and residential annexes located in the intervention blocks using Alpha cypermethrin 40 mg/m^2^.

### Outcomes

The primary outcome was the incidence of infection by *L. infantum* in the eligible population after 18 months of entering the study as determined by conversion of the MST at 18 months of follow-up (MST negative at baseline) or diagnosis of active visceral leishmaniasis.

### Randomization procedure

In order to guarantee that the four selected blocks in each of the ten selected localities would have one of the four intervention schemes, allocation was performed as follows: (a) for each locality, a number was assigned to each block, (b) the intervention schemes were ordered as described above, and (c) using the command “sample” in Stata, the first block sampled was allocated to intervention (i), the second to intervention (ii) and so on. At the end, each intervention scheme was allocated to a total of ten blocks throughout the ten selected localities.

### Sample size and power

We estimated a cumulative incidence of infection of 35% in the non-intervention group based on data from a previous intervention study in this area [20]. We calculated that a sample size of 150 persons per intervention group would give a power of 80% to detect as significant (p≤0.05) a difference of 15% in the incidence comparing non-intervention group with any of the intervention groups, taking into account an intraclass correlation coefficient of 0.03 due to the cluster sampling design. Sample size and power estimation were performed using the package “CRTSize” in R software.

### Statistical analysis

Cumulative incidence of infection in 18 months, crude risk ratios (RR) and 95% confidence intervals (95%CI) were calculated for each category of intervention scheme. To assess the adequacy of randomization, we determined the distribution of selected baseline socioeconomic and environmental characteristics and the prevalence of infection (MST positivity) by intervention category. Those variables showing a statistical difference between any of the intervention groups in comparison to the control group at a p-value ≤0.2 were selected to be adjustment variables in a multivariate analysis for assessing the effects of interventions on the incidence of infection. Chi-square and t-tests were used for categorical and continuous variables, respectively.

The effects of each type of intervention scheme on the incidence of infection were assessed by calculating RR and 95% CI using Poisson population-averaged models from generalized estimating equations with robust variance, an exchangeable correlation model, and designating each block as the clustering (panel) variable [21]. Considering that both older age and male sex have frequently been associated with VL infection in Latin America [22], [23], [24], [25], we decided to include them in the multivariate models independently of any statistical criteria. In addition, effects of interventions were controlled for the baseline prevalence of infection as assessed by the MST in each block [21].

Analyses were performed using both intent-to-treat and per-protocol approaches. Although intent-to-treat is usually a preferable approach [26], a per-protocol analysis was considered useful in this setting since one of the interventions, namely culling of infected dogs, would only occur if a dog in a block under this intervention was found to be infected (it might not have happened) and the team of health workers of the ZCC could remove the dog from the environment. Failure to remove infected dogs is not uncommon, since the infected dogs are not detected immediately in the field, but only after two tests performed at the ZCC. When returning to the field, the team might not be allowed by the owner to remove the dog, the house might be closed, or the dog might not be at home.

Statistical analyses were performed using Stata/MP, version 11.2 (STATA Corp., College Station, TX).

### Sensitivity analysis of biases

We used sensitivity analysis to explore quantitatively the likelihood of bias due to loss to follow-up. For this, we performed the same analyses described above using simple imputation of the outcome under the assumption of random missingness. This step was implemented in Stata/MP, version 11.2 (STATA Corp., College Station, TX) using the “mi” command.

### Ethical issues

This study protocol was approved by the Committee on Research Ethics of the Institute for Public Health Studies of the Federal University of Rio de Janeiro. Written informed consent was obtained from all adult subjects and from parents or legal guardians of child participants.

## Results

**Figure 2:**
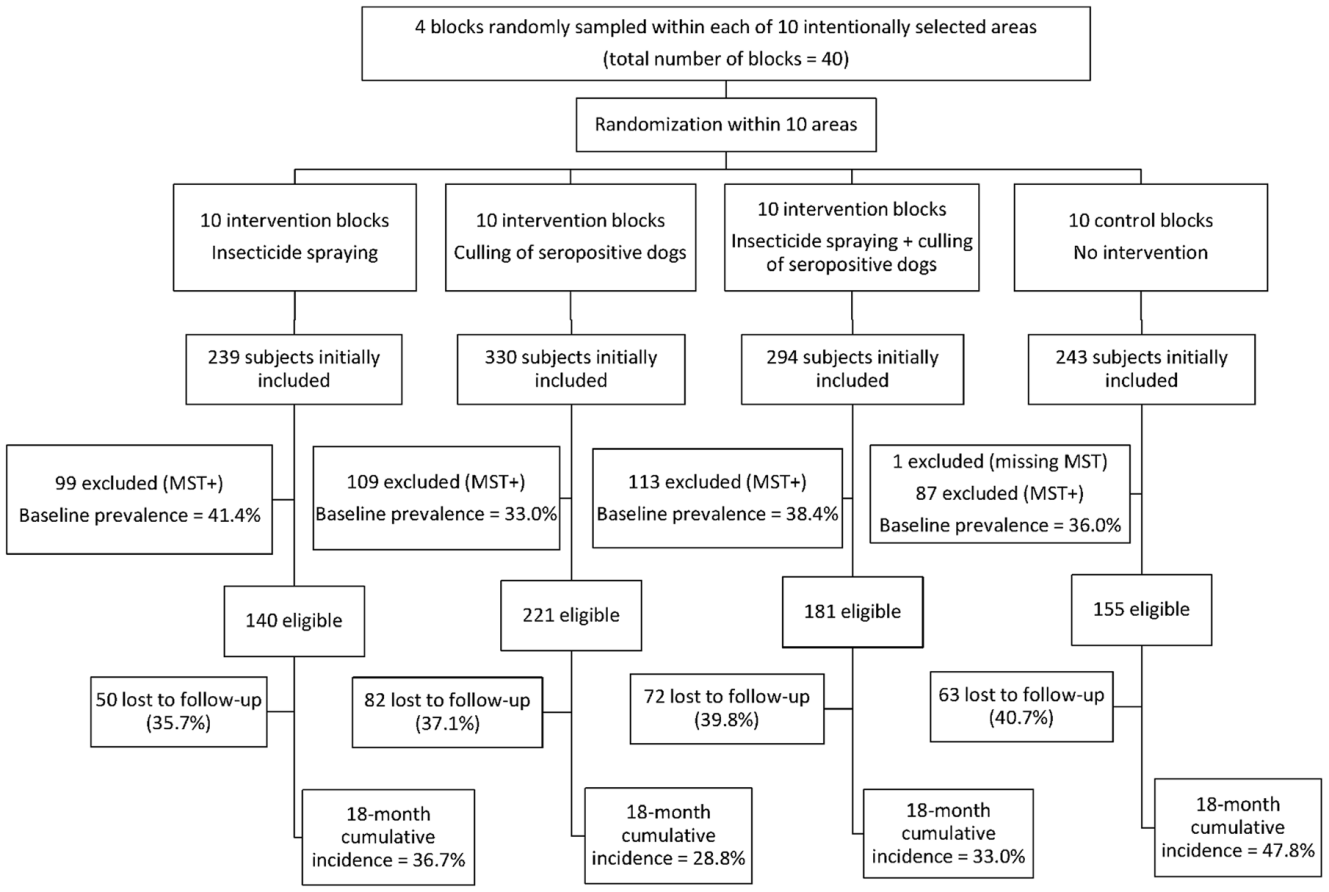
Baseline prevalence of infection, number of subjects eligible for follow-up, losses to follow-up and 18-month cumulative incidence of infection by type of intervention.

Figure 2 is a flow diagram of the study with information for each intervention arm on the number of individuals initially selected, eligible for follow-up, and lost to follow-up.

Baseline prevalence of infection varied from 33.0% to 41.4% (Figure 2), and no statistically significant difference was found comparing each intervention group with the control group (all p-values >0.2). In contrast, the 18-month cumulative incidence of infection was significantly higher in the control group as compared to the culling dog (p = 0.003) and culling dog plus vector spraying (p = 0.033) groups, but not as compared to the vector spraying group (p = 0.128). Losses to follow-up varied from 35.7% to 40.7% between intervention groups, but no statistically significant difference was found comparing each intervention group with the control group (all p-values >0.3).

Table 1 shows the distribution of selected baseline socioeconomic and environmental characteristics for each intervention group. The dog culling group showed higher mean years of living in the residence and a smaller percentage of households with a chicken shed in the peridomestic environment as compared to the control group (p = 0.015 and p = 0.046, respectively). No other statistically significant difference with any variables or groups was detected.

**Table 1:** Baseline socio-demographic and environmental characteristics by intervention groups, Teresina, Piauí, Brazil, 2004.

	Intervention groups			
	Insecticide spraying	Dog culling	Dog culling + insecticide spraying	No intervention (Control)
Variable				
Average age in years (SD)	26.1 (11.6)	29.1 (12.9)	28.9 (12.5)	27.5 (12.2)
Average years living in this residence (SD)	5.5 (5.4)	6.7 (5.7)^a^^,^ b	5.2 (5.8)	5.3 (4.8)
Male (%)	30.7	29.4	32.6	34.8
Literacy of household head less than elementary (%)	85.6^b^	83.1	81.6	79.5
History of VL in the household (%)	2.9	1.8	2.8	3.2
Owns a dog (%)	37.9	32.1	39.2	33.6
Presence of other animals in house or in the peridomestic environment (%)	53.6	50.2	48.1	49.0
Presence of a kennel in the peridomestic environment (%)	5.7	5.9	9.4^b^	4.5
Presence of a chicken shed in the peridomestic environment (%)	5.0	1.4^a^^,^ b	1.7^b^	5.2

a - statistically significant difference from the control group (p<0.05).

b - statistically significant difference from the control group (p<0.20).

In addition to sex, age and baseline prevalence of infection, the variables years of living in the residence, presence of a chicken shed in the peridomestic environment, literacy of the household head (higher in the insecticide spraying group, p = 0.168), and the presence of a kennel (more commonly found in the dog culling+insecticide spraying group, p = 0.093), were selected for multivariate analysis according to the p-value <0.2 criterion.

**Figure 3:**
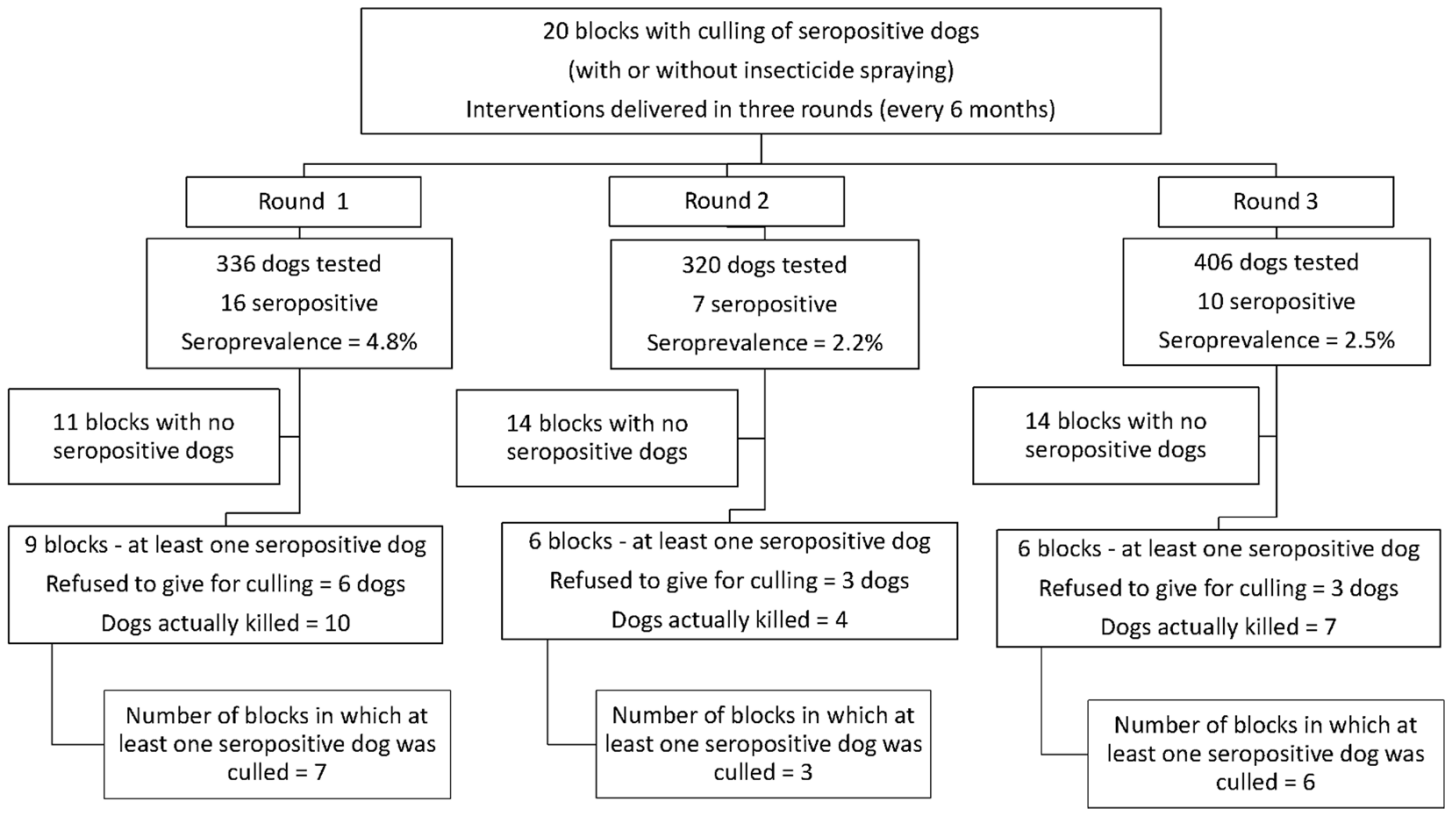
Number of dogs tested, seroprevalence and coverage of the dog culling intervention by round of delivery.

In the blocks under the dog-culling intervention (solely or combined with insecticides), a total of 3,932 houses were visited during the three intervention rounds (1,275 in the first, 1,326 in the second, and 1,331 in the third). Seven hundred and eighty houses (19.8%) harbored a total of 1,368 dogs (1.75 dogs per house with dogs). A total of 1,062 dogs (77.6%) had blood samples collected and the global prevalence of infection was 3.1% (33 seropositive dogs). Prevalence by period of intervention was 4.8% (round 1), 2.2% (round 2), and 2.5% (round 3). Among the 33 seropositive dogs, only 21 (63.6%) were removed from the environment. Owners of 12 dogs refused to give them for culling. Among the 20 blocks under dog-culling intervention, 5 (25%) did not have any seropositive dog identified and another 3 (15%) with seropositive dogs did not have them removed. In summary, only 12 (60%) of the blocks under dog-culling intervention actually experienced the removal of at least one infected dog (Figure 3).

In the 20 blocks under the insecticide-spraying intervention, a total of 3,321 houses were visited during the three intervention rounds (1,101 in the first, 1,108 in the second, and 1,112 in the third). Spraying coverage varied in each period, with 73.8% of the houses sprayed in the first, 58.0% in the second, and 67.0% in the third. The main reason for lack of universal coverage in insecticide spraying was the fact that some houses were closed or not inhabited.

Table 2 shows relative risks (RR) and respective 95% CI for the effect of interventions on the 18-month cumulative incidence of infection, using both intent-to-treat and per-protocol analyses. In all analyses, all three intervention schemes were associated with some protection, but only the dog-culling strategy alone was significantly associated with a reduction in the incidence of infection. In the intent-to-treat analysis, individuals living in blocks under such intervention had a 38% decrease in the 18-month risk of developing infection. In the per-protocol analysis, a decrease of 52% in the 18-month risk of infection was detected for individuals living in blocks under such intervention and in which at least one infected dog was detected and removed from the environment.

**Table 2:** Relative risks (RR) and respective 95% CI for the effect of interventions on the 18-month cumulative incidence of infection, using both intent-to-treat and per-protocol analyses.

	Intent-to-treat analysis					
Intervention group	RR^a^	95% CI	p-value	RR^b^	95% CI	p-value
Insecticide spraying	0.76	0.54–1.05	0.094	0.86	0.63–1.16	0.310
Dog culling	0.60	0.40–0.90	0.014	0.62	0.42–0.91	0.015
Dog culling+Insecticide spraying	0.69	0.45–1.06	0.087	0.75	0.51–1.11	0.153
Control	1.00					

	Per-protocol analysis					
Intervention group	RR^a^	95% CI	p-value	RR^b^	95% CI	p-value
Insecticide spraying	0.76	0.54–1.05	0.094	0.86	0.63–1.15	0.287
Dog culling	0.48	0.25–0.93	0.028	0.48	0.28–0.83	0.009
Dog culling+Insecticide spraying	0.63	0.39–1.02	0.061	0.69	0.43–1.10	0.119
Control	1.00					

a Crude.

b Adjusted for sex, age, baseline prevalence of infection, years of living in the residence, presence of a chicken shed in the peridomestic environment, literacy of the household head and the presence of a kennel in the peridomestic environment.

Table 3 shows the results of similar analyses as Table 2, but with imputed data on the outcome. Not only the strength of all RR estimates decreased, but also no intervention was significantly associated with a reduction in the incidence of infection. These results suggest that losses to follow-up might have introduced selection bias in the study.

**Table 3:** Relative risks (RR) and respective 95%CI for the effect of interventions on the 18-month cumulative incidence of infection, using both intent-to-treat and per-protocol analyses – sensitivity analysis of bias due to selective loss to follow-up.

	Intent-to-treat analysis					
Intervention group	RR^a^	95% CI	p-value	RR^b^	95% CI	p-value
Insecticide spraying	0.83	0.48–1.45	0.451	0.91	0.49–1.69	0.713
Dog culling	0.72	0.50–1.04	0.078	0.73	0.51–1.03	0.073
Dog culling+Insecticide spraying	0.80	0.56–1.13	0.195	0.83	0.59–1.18	0.281
Control	1.00					

	Per-protocol analysis					
Intervention group	RR^a^	95% CI	p-value	RR^b^	95% CI	p-value
Insecticide spraying	0.84	0.43–1.64	0.542	0.92	0.43–1.95	0.782
Dog culling	0.61	0.36–1.03	0.064	0.59	0.34–1.02	0.056
Dog culling+Insecticide spraying	0.70	0.36–1.37	0.250	0.77	0.38–1.55	0.390
Control	1.00					

a Crude.

b Adjusted for sex, age, baseline prevalence of infection, years of living in the residence, presence of a chicken shed in the peridomestic environment, literacy of the household head and the presence of a kennel in the peridomestic environment.

## Discussion

In this study, as in another one in the same area [20], only dog culling showed some effect on reducing the incidence of infection, although sensitivity analysis suggests that this effect might be biased due to selective loss to follow-up. In any case, estimates of the putative effectiveness of such intervention varied between 27% and 52%, depending on the type of analysis performed. A reduction of the magnitude of the incidence of infection in this range might have an effect on the incidence rates of clinical VL, but this would be probably smaller, since susceptibility for developing clinical symptoms after infection is mediated by other factors such as age, genetics and nutrition [5], [9], [10]. Indeed, a mathematical model estimated that killing 2/3 of the infected dogs, as in our study, would only lead to a reduction of the incidence of human disease by less than 20% [27].

In general, the results of this study reinforce the generally accepted idea that culling seropositive dogs and insecticide spraying, the pillars of the Brazilian VL control program for at least 50 years, are not effective strategies for interrupting the spread of the disease, at least in the way they are implemented in the country [4], [14], [15], [28]. In fact, the epidemiological situation leaves no doubt of the failure of both strategies. Disease counts have been on the rise since the 1980s, and VL is geographically spreading to areas in which it has not been reported before. From 1980 to 2010, around 80,000 cases of VL were reported in Brazil, with around 4,200 deaths. The mean number of cases reported per year increased from 1,601 (1985–1989) to 3,814 (2006–2010). In the 1990s, only 10% of cases occurred outside the Northeast region, but in 2010 the proportion reached 50% of cases. From 2009 to 2011, autochthonous cases of VL were reported in more than 20% of the municipalities and in 21 of the 27 states of the country.

The logic behind the use of such control measures in VL is the assumption that the incidence of *L. infantum* infection in humans is directly related to the number of infectious dogs and the vectorial capacity of the sand fly population to transmit infection from dogs to humans [27]. On the one hand, insecticide spraying decreases vector longevity, which is the major determinant of vectorial capacity. On the other hand, killing of infected dogs reduces the life expectancy of the reservoir population. Therefore, either vector control or dog culling theoretically can be effective [27]. However, many operational problems impair the effectiveness of these interventions such as the well-known inaccuracy of the serological tests used to identify canine infection by *L. infantum* in the field, the usual long time between identification of a seropositive dog and its removal, the fast substitution of sacrificed dogs by new, susceptible ones, insufficient knowledge about sand fly breeding sites and behavior, lack of available equipment and trained personnel for large-scale interventions, low coverage of insecticide spraying, deficiencies in quality control concerning insecticide handling, and lack of sustainability of control actions [4], [15], [20], [28], [29], [30], [31]. Considering all these problems, the low estimates of effectiveness for both interventions obtained in this study are not surprising, since it was designed to assess their effectiveness as implemented in practice. In this sense, our study provides some basis for comparisons of future studies that attempt to address the extent to which such operational problems affect the performance of interventions.

A point that deserves further attention is that not all infected dogs become infectious and the usual serological tests used in practice do not separate infectious from non-infectious animals [4], [32], [33]. Control measures targeting infectious dogs could be a more effective approach since interventions focused on just those animals that contribute mostly to transmission tend to be more efficient [33], [34], [35]. Actually, highly infectious dogs can be distinguished from non-infectious dogs adopting quantitative PCR for detecting parasite loads in ear tissue [33]. However, a control strategy oriented to remove from the environment just the highly infectious dogs might not be sufficient to interrupt transmission, since asymptomatic dogs can also transmit *Leishmania* to sandflies.

The relatively low effectiveness of dog culling (38%, considering the results from intent-to-treat analysis, a generally more accepted approach) leads to the question of the ethics of maintaining this strategy for VL control [14], [36]. In settings with low prevalence of canine infection, as in our study, the moderate specificity of the tests usually used in the field [37], [38], in particular to detect asymptomatic infection, leads to a low positive predictive value and, consequently, the sacrifice of many dogs that are actually not infected. Removing such dogs that are actually not contributing to transmission may have even an undesirable effect, since most of them will be replaced by new susceptible ones. This problem, along with the growing lack of acceptability of dog culling by the communities, makes the sacrifice of dogs an increasingly difficult control strategy to be sustained in Brazil.

In light of the low effectiveness of the dog culling strategy, a further point that might also be considered is the possibility that other reservoir mammals contribute to VL transmission in urban settings. Studies have confirmed that humans, crab-eating foxes, opossums, domestic cats, and black rats can transmit *L. infantum* to sand flies although their importance has been minimized [4]. However, in certain scenarios these secondary reservoirs could conceivably play a role in sustaining transmission, and further studies on their infectious potential are needed.

In spite of the geographic expansion and increase in number of cases of VL in Brazil in recent years, it is possible that the situation would have been even worse in the absence of these interventions. Therefore, any decision concerning the discontinuation of either of these control measures or their substitution by others, such as dog collars impregnated with insecticides, treatment of dogs, or dog vaccination, should be accompanied by a detailed monitoring of canine infection and human cases at the local level.

Several limitations of this study need to be highlighted. First, since 38% of the eligible population was lost to follow-up, selection bias is a threat to the validity of the study results. Indeed, sensitivity analysis using single imputation of the outcome, assuming random missingness, generated results compatible with non-effectiveness of all interventions evaluated. It should be noted, however, that the point estimates for the dog culling strategy still showed a protective effect of 27% (p = 0.073, intent-to-treat analysis) and 41% (p = 0.056, per-protocol analysis), making it difficult to conclude about the complete ineffectiveness of this control measure. Losses to follow-up were also common in other intervention trials in Brazil, ranging from 24% [39] to 44% in one year [20], which stresses the difficulties of performing this type of study among urban population living in deprived areas. The majority of the losses in this study were due to migration to other neighborhoods within the city, as reported by the neighbors. Second, the lack of results of serological tests at each 6 months of follow-up impaired the ability to verify the potential short-term effect of the interventions, since an antibody response to infection is built rapidly after infection [40]. Third, monitoring the effect of interventions on the incidence of VL was not possible due to the relative rarity of clinical disease. Based on incidence rates of VL in these neighborhoods from 2000 to 2002, one would have expected around 0.5 cases per 1,000 persons in a period of two years, making analysis of this outcome unfeasible in this study. This limitation needs to be taken into consideration when using the results of this study for informing decision on whether to interrupt or change the current interventions against VL, since the impact of an intervention on MST conversion and its impact on clinical VL might not be the same. Fourth, the high incidence of infection should be considered with caution, since hypersensitivity to thimerosal, used as a preservative in the Montenegro antigen, and the sensitization potential of a previous exposure to MST, might have contributed to the occurrence of false-positive cases [41], [42]. However, there is no objective reason to believe that such error would happen differentially between the intervention areas, suggesting that the estimates of effectiveness might have well been underestimated [43]. Finally, due to the high baseline prevalence of infection, high rates of loss to follow-up and a high intraclass correlation coefficient in the actual data (0.057), the final sample size provided low statistical power to detect as significant observed differences between the interventions. Indeed, power ranged from 15% (when comparing insecticide spraying alone and no intervention) to 53% (when comparing dog-culling alone and no intervention). Such variation in the statistical power occurred mainly because the number of subjects actually followed-up and the differences observed in the incidence of infection varied between areas of intervention.

Despite these limitations, this study overcomes some methodological problems of previous studies, in particular regarding the number of clusters randomly allocated to different interventions. For instance, except for one study [20], all other controlled trials in Latin America evaluating the same control measures as our study used either a before-after approach in just one area [44] or a 1∶1 or 2∶1 comparison of intervention and control areas [39], [45], [46], [47]. These approaches are inadequate for evaluating interventions, due to the high risk of lack of comparability between areas in terms of transmission intensity, but also because of the impossibility of making statistical inferences without assessing between-cluster variation [21]. A minimum of four clusters per intervention arm has been recommended for cluster randomized trials [21]. Also, some of the above studies did not evaluate the effect of interventions on human infection or disease [44], [46], which are the most appropriate outcome measures for public health purposes. Advantages of our study as compared to a previous one in the same area [20], include a larger sample size and the use of clusters not restricted to just one neighborhood, which decreases the odds of contamination between interventions in neighboring clusters [21].

In summary, the continuous spread of VL in Brazil after more than 40 years of large scale deployment of insecticide spraying and dog culling indicates an urgent need for revision of the Brazilian VL control program. While waiting for the development of an effective human vaccine, effectiveness of other control measures, such as insecticide impregnated dog collars, topical insecticides for dogs, canine vaccines, and impregnated nets for humans should be evaluated in trials using solid methodologies and powered for detecting effects of such intervention on clinical outcomes. The delivery of interventions should be modified according to the different transmission scenarios, preferably targeting the areas at highest risk. Efforts to solve operational barriers to the adequate implementation of preventive measures are paramount. Finally, a broad commitment of both scientific and civil societies is necessary to interrupt the seemingly relentless progression of VL towards becoming one of the most serious infectious diseases of Brazilian urban populations.

## Supporting Information

Figure S1 (A–H) Schematic representation of the sampling process. Schematic representation of a locality with 42 blocks (A). First block (block 30) selected at random (B). All blocks sharing a border with the first block selected (block 30) were excluded from the pool of eligible blocks for further selection (C). Second block (block 17) selected at random from the pool of eligible blocks (D). All blocks sharing a border with the second block selected (block 17) were excluded from the pool of eligible blocks for further selection (E). Third block (block 21) selected at random from the pool of eligible blocks (F). All blocks sharing a border with the third block selected (block 21) were excluded from the pool of eligible blocks for further selection (G). Finally, the fourth and last block for this locality (block 33) was selected at random from the pool of eligible blocks (H).
(TIF)

Database S1 Database with variables used in the analysis of this study. NA indicates missing data.
(XLS)
