# Supplementary figures and images for: Effectiveness of Insecticide Spraying and Culling of Dogs on the Incidence of Leishmania infantum Infection in Humans: A Cluster Randomized Trial in Teresina, Brazil
Source: PLoS Negl Trop Dis. 2014 Oct 30;8(10):e3172. doi: 10.1371/journal.pntd.0003172 (PMC4214628; doi:10.1371/journal.pntd.0003172)

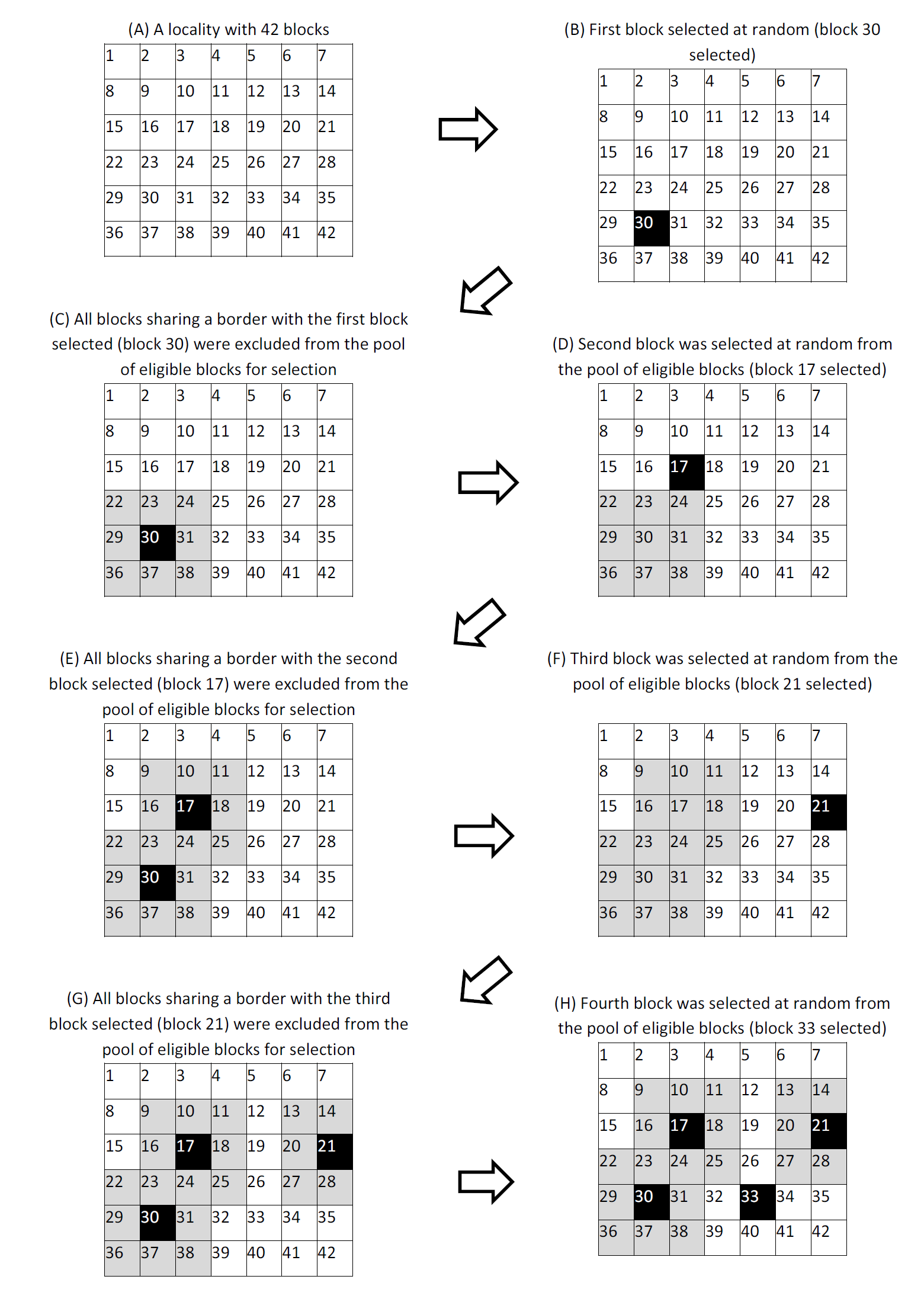

Supplement: Figure S1 — (A–H) Schematic representation of the sampling process. Schematic representation of a locality with 42 blocks (A). First block (block 30) selected at random (B). All blocks sharing a border with the first block selected (block 30) were excluded from the pool of eligible blocks for further selection (C). Second block (block 17) selected at random from the pool of eligible blocks (D). All blocks sharing a border with the second block selected (block 17) were excluded from the pool of eligible blocks for further selection (E). Third block (block 21) selected at random from the pool of eligible blocks (F). All blocks sharing a border with the third block selected (block 21) were excluded from the pool of eligible blocks for further selection (G). Finally, the fourth and last block for this locality (block 33) was selected at random from the pool of eligible blocks (H). (TIF) [file pntd.0003172.s001.tif]
